# Conservation of physiological dysregulation signatures of aging across primates

**DOI:** 10.1111/acel.12925

**Published:** 2019-02-11

**Authors:** Gabriel Dansereau, Tina W. Wey, Véronique Legault, Marie A. Brunet, Joseph W. Kemnitz, Luigi Ferrucci, Alan A. Cohen

**Affiliations:** ^1^ Groupe de recherche PRIMUS, Department of Family Medicine University of Sherbrooke Sherbrooke Quebec Canada; ^2^ Department of Biochemistry University of Sherbrooke Sherbrooke Quebec Canada; ^3^ PROTEO Quebec Network for Research on Protein Function, Structure, and Engineering Quebec Canada; ^4^ Department of Cell and Regenerative Biology University of Wisconsin School of Medicine and Public Health Madison Wisconsin; ^5^ Wisconsin National Primate Research Center University of Wisconsin‐Madison Madison Wisconsin; ^6^ Translational Gerontology Branch, Longitudinal Studies Section National Institute on Aging, National Institutes of Health, MedStar Harbor Hospital Baltimore Maryland

**Keywords:** aging, biomarkers, dysregulation, homeostasis, Mahalanobis distance, nonhuman primates

## Abstract

Two major goals in the current biology of aging are to identify general mechanisms underlying the aging process and to explain species differences in aging. Recent research in humans suggests that one important driver of aging is dysregulation, the progressive loss of homeostasis in complex biological networks. Yet, there is a lack of comparative data for this hypothesis, and we do not know whether dysregulation is widely associated with aging or how well signals of homeostasis are conserved. To address this knowledge gap, we use unusually detailed longitudinal biomarker data from 10 species of nonhuman primates housed in research centers and data from two human populations to test the hypotheses that (a) greater dysregulation is associated with aging across primates and (b) physiological states characterizing homeostasis are conserved across primates to degrees associated with phylogenetic proximity. To evaluate dysregulation, we employed a multivariate distance measure, calculated from sets of biomarkers, that is associated with aging and mortality in human populations. Dysregulation scores positively correlated with age and risk of mortality in most nonhuman primates studied, and signals of homeostatic state were significantly conserved across species, declining with phylogenetic distance. Our study provides the first broad demonstration of physiological dysregulation associated with aging and mortality risk in multiple nonhuman primates. Our results also imply that emergent signals of homeostasis are evolutionarily conserved, although with notable variation among species, and suggest promising directions for future comparative studies on dysregulation and the aging process.

## 1 INTRODUCTION

Two outstanding questions in the biology of aging are (a) What general biological framework can integrate the diversity of physiological mechanisms underlying aging (Cohen, 2017a)? and (b) How and why do aging and longevity vary across species (Cohen, 2017b; Jones et al., 2014)? Recent research in humans has emphasized that aging is a product of complex system dynamics, rather than the sum of isolated mechanisms, leading to increased interest in physiological networks and feedback among different systems (Cohen, Martin, Wingfield, McWilliams, & Dunne, 2012; Fried et al., 2009; Han et al., 2017; Hoffman et al., 2014; Kriete, Bosl, & Booker, 2010). Accordingly, some analyses have moved from examining single candidate biomarkers of aging in isolation to multivariate approaches that address interrelated system functioning and dynamics and more directly test hypotheses about emergent processes. One process proposed to be an important driver of aging is physiological dysregulation, the progressive loss of homeostasis in complex biological networks. In this scenario, the consequences of aging largely result from system‐level breakdown of regulation, rather than problems in single mechanisms, such as gene expression or oxidative stress (Cohen et al., 2012). A number of studies in humans now support this hypothesis, showing that dysregulation can increase with age and predict increased mortality or other health risks (Arbeev et al., 2016; Cohen et al., 2013; Crimmins, Johnston, Hayward, & Seeman, 2003; Fried et al., 2009; Glei, Goldman, Chuang, & Weinstein, 2007; Karlamangla, Singer, & Seeman, 2006; Yang & Kozloski, 2011).

The vast majority of research on dysregulation and its association with aging has been conducted in humans, so we do not know whether system dysregulation is widely associated with aging, what health impacts it has in other animals, or whether signals of homeostasis are conserved across species. This also limits the identification of potential model nonhuman species to study dysregulation. Studies on related concepts in stress physiology hint that physiological dysregulation and its effects are taxonomically widespread. For example, allostatic load is the cumulative negative effects of stress response activation to maintain homeostasis and increasing dysregulation of physiological systems implicitly underlies these negative effects (McEwen, 1998; McEwen & Wingfield, 2003). In birds, higher allostatic load can be associated with biomarkers of aging (Hau et al., 2015) and negative physiological effects linked to greater predation risk (Travers, Clinchy, Zanette, Boonstra, & Williams, 2010). However, these studies focusing on the stress response typically do not examine multiple physiological systems simultaneously or focus on dysregulation per se.

An additional challenge is to develop appropriate and general measures of physiological dysregulation. One recently proposed measure (Cohen et al., 2013) uses Mahalanobis distance (*D_M_*) (Mahalanobis, 1936), a multivariate distance measure, to incorporate biomarker correlation structure. *D_M_* reflects the aberrance of an observation (an individual's biomarker profile) from the multivariate mean of a reference population, which represents a “normal” or homeostatic physiological state. The reference population is often the study population itself, under the assumption that the mean physiological state approximates the optimal state. Using the joint distribution of many markers allows us to incorporate different combinations of biomarkers levels into an aggregate score, and higher *D_M_* scores can result from both very unusual values for biomarkers, whether higher or lower than average, and unusual combinations of biomarker values. For example, if statistical distance was calculated for height and weight of people, a person who was both much taller than average and much lighter than average would have a higher score than someone who was taller and heavier than average. Under a complex system perspective (Cohen, 2016), larger deviations from the mean distribution of physiological measurements indicate loss of homeostasis, that is, greater dysregulation, and we expect simultaneous dysregulation in multiple systems to result in higher *D_M_* scores. *D_M_* increases with age and predicts mortality and health risks in different human populations using biomarkers from diverse physiological systems (Arbeev et al., 2016; Cohen, Legault, Li, Fried, & Ferrucci, 2018; Cohen et al., 2013; Li et al., 2015). Furthermore, it appears to be robust to variation in biomarker composition and study populations, indicating the importance of an emergent quality rather than of specific biomarkers (Cohen et al., 2015). Another study used *D_M_* to measure body condition in shorebirds and showed that higher *D_M_* was associated with poor health and performance outcomes (Milot et al., 2014). These studies suggest *D_M_* may be used to examine dysregulation across species.

Nonhuman primates (NHPs) provide valuable model and comparative systems for research on aging and physiology (Bronikowski et al., 2011; Colman & Kemnitz, 1998; Lane, 2000; Muntané et al., 2018). In particular, NHPs share more fundamental biological features with humans than other common model species, such as rodents or invertebrates, and as such are likely to be better models of complex emergent processes. Studies on NHPs have provided insights into primate aging and potential interventions. For example, rhesus macaques (*Macaca mulatta*) exhibit many age‐related changes in physiological parameters similar to humans (Maestripieri & Hoffman, 2011; Smucny et al., 2001) and were the first primates in which caloric restriction was demonstrated to delay age‐related illness and mortality (Colman et al., 2009). Recent calls have been made for more research on effects of allostatic load on health and aging in primates (Edes & Crews, 2017; Maestripieri & Hoffman, 2011), and some work has taken advantage of the primate group to investigate evolutionary origins of senescence (Bronikowski et al., 2011) and genetic mechanisms of aging (de Magalhães & Church, 2007; McLain & Faulk, 2018). Yet, the great majority of aging studies in NHPs have focused on a few commonly studied species rather than cross‐species comparisons and none have focused on dysregulation per se.

Here, we take advantage of unusually detailed data on physiological biomarkers on multiple nonhuman primate species as well as two human populations to test two hypotheses: (a) that dysregulation is widely implicated in aging and poor health outcomes and (b) that homeostatic state is taxonomically conserved, to degrees correlated with phylogenetic distance. We asked whether dysregulation (measured as *D_M_* score) increased with age or differed between sexes and whether greater dysregulation predicted increased mortality risk or poor condition. We further examined how choice of biomarkers and reference population (to define healthy profiles and homeostasis) affected *D_M_*, and whether species similarities in *D_M_* correlated with phylogenetic proximity. In most NHPs studied, dysregulation was positively associated with age or health outcomes in ways similar to humans, but the exact role of dysregulation likely differs among species, and the ability to detect patterns might be limited by sampling of individuals and biomarkers. We also find that emergent homeostatic state is substantially conserved across species and correlated with phylogenetic distance.

## 2 RESULTS

Biomarker data came from long‐term human datasets and systematic longitudinal measures of nonexperimental NHPs in research centers. The 10 NHP species in this study spanned a range of taxa and expected maximum lifespans (Table 1). To measure *D_M_*, we strove to maximize the number and diversity of biomarkers to capture multiple systems, while maintaining sufficient sample sizes (Supporting information Table S1). The number of biomarkers used varied among species, ranging from 10 to 24. To check the effect of biomarker availability/choice, we replicated all analyses in two sets of data: Set 1 used variable biomarkers (10–24) depending on availability for 11 species (humans and 10 NHPs) and Set 2 used 12 fixed biomarkers (Supporting information Tables S1 & S2) across 10 species (humans and 9 NHPs). Set 2 excluded the species with the fewest available biomarkers. Thus, Set 1 provides a better representation of biomarkers, but the calculation of *D_M_* is more species‐specific. For models of aging and health risks, we calculated *D_M_* using the first (i.e., the youngest) observations for each individual of each species as the reference population for itself, while for cross‐species comparisons, we compared *D_M_* scores calculated from different possible reference populations.

**Table 1:** Primate species information

Common Name	Scientific Name	Abbreviation	Obs	IDs	Markers	Species group	Lifespan (years)^a^	Age range (years)^b^
Humans	*Homo sapiens*	Human	5,936	2,463	24	Humans	122.5	21.3 – 101.0
Chimpanzee	*Pan troglodytes*	Chimp	3,942	451	21	Great Apes	59.4	9.6 – 57.9
Orangutan	*Pongo pygmaeus*	Orang	186	30	21	Great Apes	59.0	7.1 – 40.9
Rhesus Macaque	*Macaca mulatta*	Rhesus	752	200	20	Old World Monkeys	40.0	5.6 – 33.4
Pig‐tailed Macaque	*Macaca nemestrina*	Pigtail	708	122	15	Old World Monkeys	37.6	8.1 – 32.6
Squirrel Monkey	*Saimiri sciureus*	Squirrel	391	26	19	New World Monkeys	30.2	5.1 – 25.9
Cotton‐top Tamarin	*Saguinus oedipus*	Cottontop	799	180	10	New World Monkeys	26.2	1.6 – 12.9
Common Marmoset	*Callithrix jacchus*	Marmoset	78	77	22	New World Monkeys	22.8	1.6 – 9.4
Coquerel's Sifaka	*Propithecus coquereli*	Sifaka	125	39	21	Lemurs	31.0^c^	2.6 – 26.6
Ring‐tailed Lemur	*Lemur catta*	Ringtail	174	60	22	Lemurs	37.3	2.6 – 24.1
Red‐collared Brown Lemur	*Eulemur collaris*	Rcb	107	25	22	Lemurs	35.5^d^	2.9 – 24.4

a Confirmed maximum records from AnAge (http://genomics.senescence.info/species/)

b Age ranges represented in the data

c Taken from records for Verreaux's sifaka (*Propithecus verreauxi*)

d Taken from records for brown lemur (*Propithecus verreauxi*)

### 2.1 Age and sex differences in dysregulation

**Figure 1:**
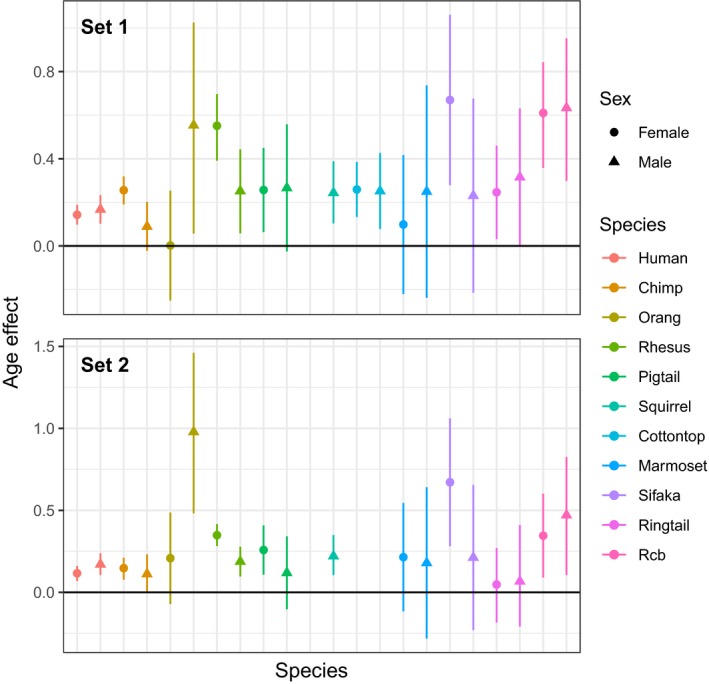
Estimated effects of age on *D_M_*, shown separately by species and sex. *D_M_* and age were centered to 0 and scaled to 1 standard deviation. Thus, the coefficient reflects the estimated effect at the mean age, and effect sizes can be compared across species. Error bars represent 95% confidence intervals. Note that this is a graphical representation of separate effects by sex, but significance of effects is interpreted from full model results (see Table S3)

We ran multilevel models (MMs) separately for each species, with fixed effects of age at observation, sex, and their two‐way interaction, with the exception of squirrel monkeys, for which we only had data on males and hence only modeled the fixed effect of age. MMs included random intercepts for individual (ID) and population for species with repeated observations on IDs (all species except common marmosets) or multiple research populations (humans, chimpanzees, rhesus macaques), respectively. *D_M_* increased with age in most species in both Set 1 and Set 2 (Figure 1; Supporting information Table S3), with only common marmosets showing no significant increase in either sex in either set. *D_M_* increased with age in both sexes in humans, chimpanzees, rhesus macaques, pig‐tailed macaques, cotton‐top tamarins, Coquerel's sifaka, ring‐tailed lemurs (only in Set 1), and red‐collared brown lemurs. *D_M_* only increased with age in males in orangutans and squirrel monkeys (for which we only had males), and it increased more slowly in males than females in chimpanzees (only in Set 1), rhesus macaques, and Coquerel's sifaka (only in Set 2). Additionally, *D_M_* was higher at the mean age in males in humans, orangutans (only in Set 2), and Coquerel's sifaka (only in Set 2), and it was lower in males in rhesus macaques (only in Set 1). Note that we present results here with unadjusted p‐values but discuss implications of multiple testing in Discussion.

### 2.2 Mortality and body condition

For NHPs with data on survival and *D_M_* scores (chimpanzees, rhesus macaques, pig‐tailed macaques, common marmosets), we ran Cox proportional hazards (PH) models with fixed effects of *D_M_*, sex, and their two‐way interaction on risk of mortality. For species with animals from multiple research centers (chimpanzees and rhesus macaques), we also included a random effect for center. Mortality risk increased strongly with higher *D_M_* in chimpanzees, pig‐tailed macaques, and common marmosets, with each unit of *D_M_* adding >50% increase in risk of mortality for these species in Set 1 and >25% increase in risk in Set 2 (Table 2). Additionally, male chimpanzees had over twice the risk of mortality as females in both sets, and male rhesus macaques had lower risk of mortality in Set 1 (Table 2).

**Table 2:** Estimated fixed effects from Cox models of the effect of *D_M_* and sex on risk of mortality in Sets 1 and 2

	Species	IDs	Deaths	*D_M_* HR	Sex HR
1	Chimp	393	69	**1.61 (1.39, 1.87)**	**2.64 (1.57, 4.44)**
	Rhesus	113	98	1.04 (0.88, 1.23)	**0.67 (0.47, 0.95)**
	Pigtail	72	70	**1.56 (1.16, 1.98)**	0.85 (0.49, 1.48)
	Marmoset	72	52	**1.65 (1.13, 1.93)**	0.84 (0.48, 1.46)
2	Chimp	400	72	**1.49 (1.28, 1.75)**	**2.47 (1.49, 4.10)**
	Rhesus	198	148	0.99 (0.86, 1.15)	0.74 (0.53, 1.03)
	Pigtail	99	97	**1.27 (1.05, 1.55)**	1.05 (0.69, 1.60)
	Marmoset	78	57	**1.52 (1.20, 1.92)**	0.86 (0.50, 1.48)

Note

HR, hazard ratio.

*D_M_* was centered to 0 and scaled to 1 standard deviation, within species, to facilitate comparison among species. 95% confidence intervals are shown in parentheses. The reference sex is female. Significant effects are in bold.

Unintentional weight loss in the elderly can be associated with morbidity and mortality (Alibhai, Greenwood, & Payette, 2005). We analyzed body mass changes in NHPs and found that mass decreased in old age in all species and faster declines predicted higher risk of mortality regardless of age in several species (Supporting information Tables S4 & S5). Therefore, declining mass seems to reflect poor condition associated with old age and reduced survival in NHPs. We modeled the effect of *D_M_* on current mass in six NHPs with data on both mass and *D_M_* (chimpanzees, rhesus macaques, squirrel monkeys, Coquerel's sifakas, ring‐tailed lemurs, and red‐collared brown lemurs), and we modeled the effect of *D_M_* on subsequent mass change in four species (chimpanzees, rhesus macaques, Coquerel's sifakas, and red‐collared brown lemurs). We found no associations between *D_M_* and current mass (Table 3) or subsequent decrease in mass in any species (all *p* > 0.05).

**Table 3:** Estimated fixed effects from multilevel models of association between *D_M_* and body mass in Sets 1 and 2

	Species	Obs	IDs	*D_M_*
1	Chimp	1,484	286	−0.19 (−0.44, 0.06)
	Rhesus	519	116	−0.03 (−0.13, 0.06)
	Squirrel	343	26	<0.01 (−0.01, 0.01)
	Sifaka	92	31	0.01 (−0.01, 0.03)
	Ringtail	115	49	<0.01 (−0.03, 0.01)
	Rcb^a^	80	24	−0.01 (−0.01, 0.02)
2	Chimp	1,660	304	−0.20 (−0.45, 0.05)
	Rhesus	2,096	130	0.02 (−0.03, 0.06)
	Squirrel	357	26	<0.01 (0.00, 0.01)
	Sifaka	96	31	−0.01 (−0.03, <0.01)
	Ringtail	118	49	−0.01 (−0.05, 0.03)
	Rcb^a^	84	24	−0.03 (−0.07, 0.02)

Note

Dependent variable is mass at observation. *D_M_* is centered to 0 and scaled to 1 standard deviation, within species, to facilitate comparison among species. 95% confidence intervals are shown in parentheses. Significant effects are in bold. Models controlled for age and sex effects (see, Supporting information Table S6).

a Log‐transformed

### 2.3 Phylogeny and conservation of homeostasis

To examine conservation of homeostasis and dysregulation signatures across species, we calculated *D_M_* scores from different reference populations and interpreted correlations among the different scores as indicators of similarity in underlying physiology. Higher correlations would suggest similar dysregulation patterns, while lower correlations would suggest underlying population differences in the joint distribution of biomarkers. We considered three possible reference populations: each species as a reference for itself (as in the above analyses), a common reference for all species using combined observations from all species, or one species as a reference for all species.

**Figure 2:**
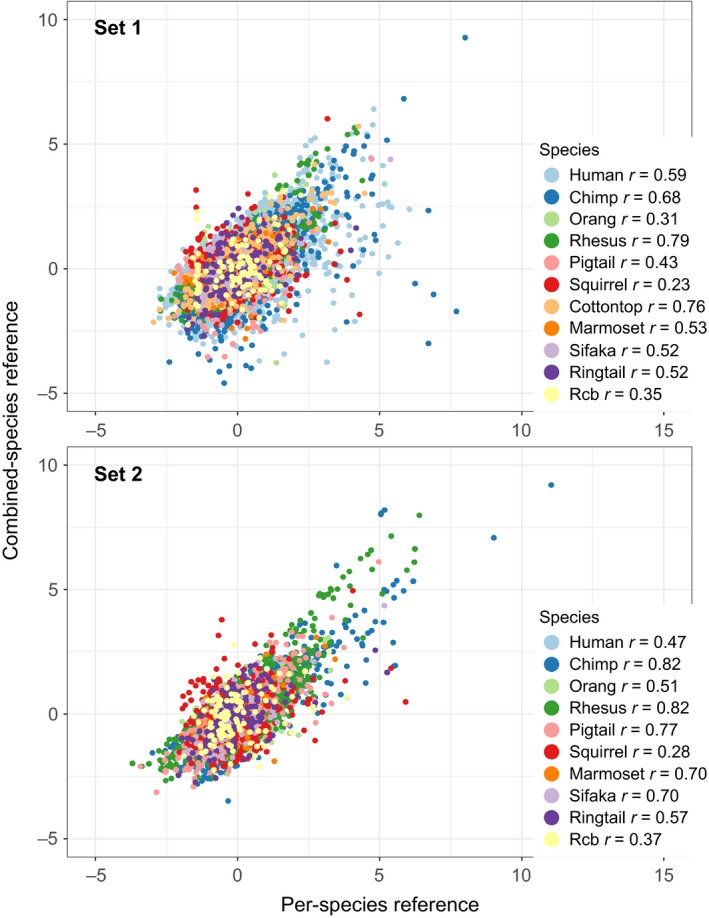
Correlation between *D_M_* scores calculated using each species as a reference for itself and *D_M_* scores calculated using combined‐species data as a reference population: Set 1 using variable numbers of biomarkers for 11 species and Set 2 using the same 12 biomarkers for 10 species. *D_M_* was centered to 0 and scaled to 1 standard deviation to facilitate comparison among species. *r* = Pearson's correlation coefficients between the two *D_M_* scores for each species (all *p* < 0.001)

**Figure 3:**
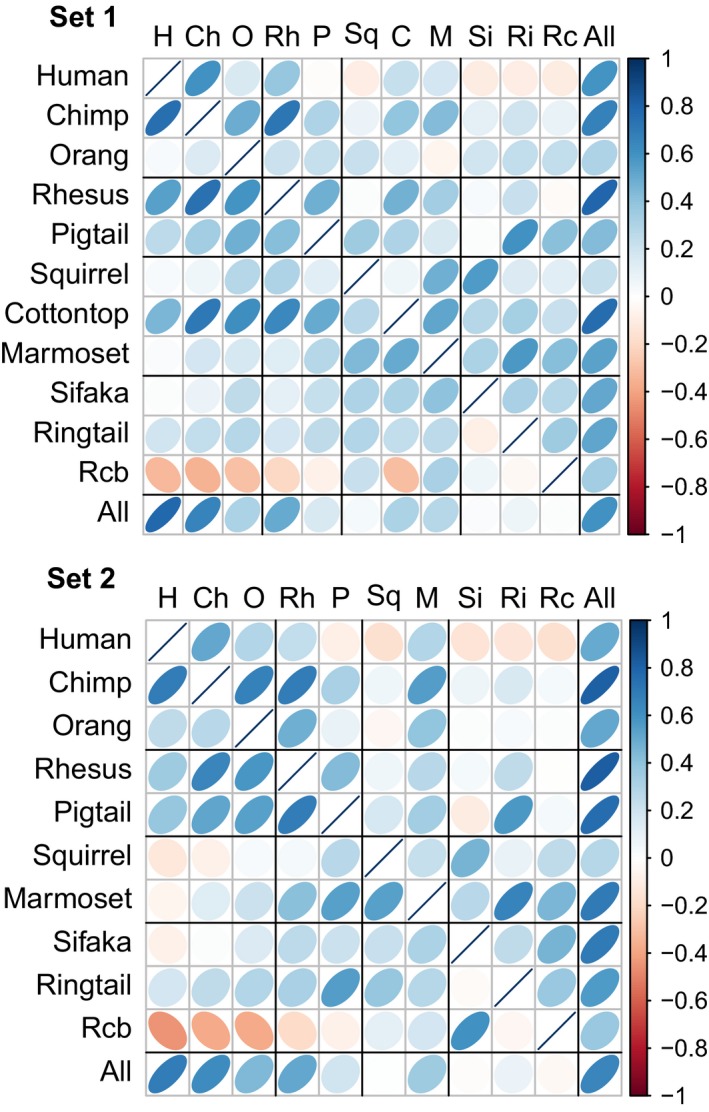
Correlation matrices for correlations (*r*) between *D_M_* scores calculated using each species as a reference for itself and using each species as the reference for the others: Set 1 using variable numbers of biomarkers for 11 species and Set 2 using the same 12 biomarkers for 10 species. Rows are species for which *D_M_* is calculated; columns are species used as the reference. The combined‐species population is presented in the last column and row for visual reference but was not used in calculating *r* between phylogeny and *D_M_*. Each cell visually represents *r* between *D_M_* scores for the row species calculated using itself or the column species as the reference. Positive *r* values are in blue and tilt to the right; negative values are in red and tilt to the left. Color intensity and ellipse eccentricity scale to the strength of *r*, that is, weak correlations appear as faint boxes, and strong correlations appear as dark, thin ellipses. Matrix diagonals represent perfect correlation (*r = *1), except for the bottom‐right cell, where the combined reference (last column) was a random subsample of the combined population (last row). Species are ordered by phylogenetic proximity to humans. Black interior lines indicate divisions between general species groupings from Table 1

There was a range of low to high positive correlations (0.23 ≤ *r *≤ 0.82, all *p* < 0.05) between *D_M_* scores obtained from using each species as its own reference and from the combined data of all species as the common reference (Figure 2), meaning that we obtained relatively similar *D_M_* values whether using species‐specific or a pooled‐species reference population. Correlations were slightly higher in Set 2 (0.28 ≤ *r* ≤ 0.82) than in Set 1 (0.23 ≤ *r* ≤ 0.79). The strength of correlation between *D_M_* scores when each species was its own reference population and scores when each species was the reference population for all other species varied among pairs of species (Figure 3). More closely related species were better references for each other, as indicated by a significant correlation between phylogenetic proximity and *r* values of *D_M_* scores calculated with different reference populations (Spearman's *ρ *= 0.327 or 0.422 (*p* < 0.001) for Sets 1 and 2, respectively).

## 3 DISCUSSION

Our key findings are twofold. First, physiological dysregulation tends to increase with age and/or predicts mortality risk across diverse primate species. This confirms a role for dysregulation in a broader phylogenetic context, although with substantial nuances and among‐species variation. Second, homeostatic signature is overall moderately conserved across primate species, declining with phylogenetic distance. This is striking given the population differences in homeostatic signature even within humans (Cohen et al., 2018, 2015), such that care is needed when using one population as a reference for another. Here, dysregulation scores calibrated within a focal species correlated well with scores obtained from pooling all species in a joint reference population, or, in some cases, even with scores calibrated on a different species. Moreover, the conserved pattern appears to be an emergent phenomenon of the system not directly predicted by its parts. This study provides the first broad evidence linking physiological dysregulation to aging and mortality across different species, and it illustrates the importance of using species comparisons to test the generality of the pattern.

In almost all of the primates studied here, dysregulation was associated with increasing age or mortality risk, but the patterns varied significantly. In most species, including humans, dysregulation increased with age, corroborating general results from previous human studies (Arbeev et al., 2016; Cohen et al., 2018), despite differences in biomarkers and age ranges used. Our multilevel models used longitudinal data with repeated observations on individuals, suggesting that dysregulation is not just correlated with age but increases with age within individuals. Exact results varied slightly across the two biomarker sets, and common marmosets never showed an association with age, so increased dysregulation appears to be a very common but not universal pattern of aging in primates. We note, however, potential limits to detecting a universal pattern due to low power in poorly sampled species and lower numbers of biomarkers in Set 2. Indeed, common marmosets were the species with the fewest observations. Prior studies noted that the predictive power of *D_M_* increased with more biomarkers (Cohen et al., 2014, 2013) and that the importance of exact biomarkers varied among populations (Cohen et al., 2014). Nonetheless, the overall strong pattern of increase in *D_M_* with age provides strong evidence that physiological dysregulation plays a fundamental role in aging across primates.

Patterns of dysregulation also varied within species. As in other work (Cohen et al., 2018), human males had higher *D_M_* than females. In several NHPs, sex differences in *D_M_* varied with age (i.e., rates of increase differed between sexes), usually with males showing a slower rate of increase, but with males showing a higher rate of increase in orangutans. A sex difference in rate of change was observed in some human studies (Arbeev et al., 2016) but not others (Cohen et al., 2018). Sex differences in dysregulation could be confounded with sex differences in the biomarkers used, especially if one sex contributes more to the reference population than the other. However, more detailed examinations of sex differences in dysregulation in humans (Cohen et al., 2018, 2015) suggest that relative dysregulation scores are stable, even if one sex is used as the reference for the other, and that the sex differences can reflect real differences in dysregulation and not just differences in biomarker values. While we did not extensively examine within‐species differences in this study, our analyses indicated consistent differences in dysregulation scores among individuals and sometimes among populations. Future research could explore if degree or rate of dysregulation corresponds to environmental factors or species’ characteristics, such as lifespans, body sizes, life histories, or social systems.

Dysregulation was associated with mortality risk in some species, although, interestingly, not always in the same species that showed associations with age. In three NHP species of the four examined, greater dysregulation predicted greater risk of mortality, and individuals in poorer condition also had higher *D_M_* scores in some NHPs. Our results concur with findings in other species (Arbeev et al., 2016; Cohen et al., 2018; Milot et al., 2014) and broadly support the hypothesis that the breakdown of physiological regulatory networks reduces longevity and health (Cohen, 2016; Fried et al., 2009; McEwen, 1998). Notably, the predicted effect of *D_M_* on survival (hazard ratio (HR) per unit *D_M_* ranging from 1.27 to 1.65) was quite large for these animals, which come from controlled environments with relatively few external sources of variation. In chimpanzees, males had much higher mortality risk than females (HR > 2.47), similar to trends in humans, while in rhesus macaques, males had lower risk. Our results also show that dysregulation can be associated with age or mortality risk independently of each other. Rhesus macaques showed an increase in *D_M_* with age but no association with survival. Conversely, common marmosets showed no increase in *D_M_* with age but had increased mortality risk. Contrary to our expectations, dysregulation was not associated with changes in body mass in any species. However, our hypothesis was that higher dysregulation would be associated with poor body condition, and body mass is affected by many factors and not a precise proxy of body condition. Additionally, we had limited ability to focus on older individuals, and loss of body condition might be a better indicator of poor health at older ages.

What does the conservation of homeostatic signature imply? On the one hand, in a complex system with interdependent components, many aspects of network configuration could be conserved by evolutionary change acting not on independent components but along key canalized axes or modules (Cohen et al., 2012). Conserved signatures of homeostatic regulation might then be expected unless there was strong divergent selection among primate lineages on particular physiological systems. Nevertheless, from a statistical perspective, a common multivariate signal is still notable as levels of individual biomarkers can vary substantially across species (Supporting information Table S2, Figure S1) and vary within species based on condition, age, sex, and other factors. It would not have been surprising if species differences in reference populations induced major changes in dysregulation rankings, yet we detected the conserved signal even with a relatively modest list of biomarkers. Evolutionary relationships also explain some, but certainly not all, of the similarity in dysregulation profiles. Species that diverged more recently tended to be better references for each other, but the correlation was only moderate.

Human *D_M_* scores were generally poorly predicted by other species. They did not have high correlations with scores from a common reference and often showed weak negative correlations with scores from nonhuman references (Figure 3). Chimpanzees were the best nonhuman reference species for humans, while rhesus macaques, which are more practical as research models, appear to provide a moderate reference. More distantly related primate species would likely be poor models of the dysregulation process in humans. Humans potentially show a more divergent pattern of dysregulation (and homeostasis), but in this dataset, human subjects would also have experienced more variable environments than NHPs, which lived in controlled conditions with regulated diets. Note that, with the combined‐species reference, distance is measured from a joint mean rather than a species‐specific mean. This may give the impression that some species are more dysregulated than others, but could instead be an artifact of small, possibly adaptive differences in mean values rather than poor average health state in some species. Indeed, the combined reference tended to perform better for individual species than any single species for another species, but was itself not particularly well predicted by any single species (Figure 3). Differences in species *D_M_* scores in this dataset might also reflect differences in response to captivity. Comparisons with patterns in free‐ranging animals or natural populations, and in general, more species comparisons at multiple taxonomic levels, will help distinguish the roles of evolution and environment in driving these species differences (Cohen, 2017a).

Some other considerations in our study should be noted. We included data on adults of all ages, thus representing a larger age range than studies of dysregulation in humans, which tended to focus on older populations. The fact that we recovered many links with age and mortality risk suggests a role of dysregulation throughout life and not just at old ages. We also modeled the linear relationship between *D_M_* and age, but preliminary analyses suggested that these could be nonlinear. Cohen et al. ()^2018^, ^2013^ noted that the relationship between age and *D_M_* in humans is likely nonlinear but was reasonably approximated by a linear relationship for their study. As some species have smaller age ranges represented and particularly fewer very old individuals, our ability to focus on older animals and model nonlinear relationships was limited by sample sizes, and linear relationships have the advantage of being simpler to compare across species. Still, there is certain to be more diversity among species in this relationship than captured in this study. Some factors could add noise to our dataset and make our results more conservative. Animal husbandry and data collection methods inevitably vary among research centers. In the calculation of *D_M_*, it was not possible to optimize transformations for all biomarkers for all species, and data are not perfectly multivariate normal. Correlations were slightly higher for most species when biomarkers were standardized among species (Figure 2), implying that standardization could be desirable for direct comparisons, where possible. Yet, in light of all these considerations, the generality of the dysregulation signal we detected is even more striking. A final point is that we presented standard *p*‐values rather than adjusting for multiple comparisons when testing hypotheses in different species and sets. Rather than changing the *p*‐value threshold, we prefer to note that, at the level of *p* = 0.05, we might expect 1 false positive out of 20 tests, and this should always be kept in mind, for example, when looking at Figure 1, which has 40 tests of association between age and *D_M_*.

In summary, multivariate measures such as *D_M_* provide insight when used to quantify emergent processes across species. Broadly speaking, there were significant links between dysregulation and aging or negative health outcomes in diverse primates, as well as significant species variation that likely emerges from both underlying species differences and variation in statistical power. Moreover, homeostatic state shows substantial conservation within the primate group over its 50–60 million year history. Our study represents an important extension of previous work in humans to NHPs, a test of the validity of primate models for human aging, and a step toward better understanding the broad biological role of physiological dysregulation.

## 4 EXPERIMENTAL PROCEDURES

### 4.1 Datasets and biomarker selection

NHP biomarker data came from the Internet Primate Aging Database (iPAD, http://ipad.primate.wisc.edu/) (Smucny et al., 2001), maintained by the University of Wisconsin‐Madison National Primate Center. All observations came from nonexperimental captive animals considered healthy at the time of sampling, and research centers contributing to iPAD were approved and accredited by the relevant oversight bodies (USDA, AAALAC, EU Directives). We chose study species based on numbers observations and biomarkers available. Human biomarker data came from two longitudinal datasets: the Baltimore Longitudinal Study of Aging (BLSA) (Ferrucci, 2008; Shock et al., 1984) and Invecchiare in Chianti (InCHIANTI) (Ferrucci et al., 2000). Initial approval for human research came from ethics committees at the respective institutions responsible for data collection, and secondary analysis was approved by the Comité d’éthique de la recherche sur l'humain du CHUS (project #14‐059). We removed observations for each species that were biologically improbable based on previously published values or visible outliers outside of recorded ranges. We only analyzed data on adults (sexually mature individuals), as determined from maturation ages for each species as listed on AnAge: the Animal Ageing and Longevity Database (http://genomics.senescence.info/species/(Tacutu et al., 2017).

### 4.2 Calculation of D_M_ scores

Mahalanobis distance (*D_M_*) is calculated as:

$D_{M}=\sqrt{{\left(x-\mu \right)}^{T}S^{-1}\left(x-\mu \right)}$, where *x* is a vector of simultaneously observed values for the variables of interest (e.g., biomarker values for a given individual at one sampling point), μ is the equal‐length vector of reference population means for each variable, and *S* is the reference population variance–covariance matrix for the variables. A *D_M_* score reflects an individual profile's deviation from the multivariate mean (which has statistical distance of 0), and higher *D_M_* scores can result from both unusual biomarker values, whether higher or lower than average ranges, and unusual combinations of biomarker values. We use this score of biomarker deviation to represent overall multisystem physiological dysregulation. More details of this measure and its calculation are available in previous studies (Cohen et al., 2018, 2013). Because of the assumption of multivariate normality, we natural log‐ or square‐root‐transformed biomarker values as needed to a best approximation of normality in the reference population. All variables were centered to 0 and standardized by subtracting the mean and dividing by the standard deviation of the reference population. Transformation and standardization of variables are specific to each reference population and thus varied slightly between sets. In our study, *D_M_* scores were never strongly correlated with individual markers (maximum *r* = 0.166, most *r* < 0.1), indicating that no single marker would strongly drive *D_M_* scores and that it was successfully capturing an emergent property.

In analyses where we used each species as its own reference or as the reference for all species, we used the first observation (i.e., the youngest visit) of each individual in the reference populations (as in Cohen et al., 2013). This approximates using a younger and presumably healthier population to generate the population mean. In calculations using a common reference population, to avoid the most well sampled species driving patterns, we randomly sampled each species for the minimum number of observations for any species (IDs = 30 in orangutans) to use in a common reference and checked that results from different samples were qualitatively similar.

### 4.3 Age and sex differences in dysregulation

We initially ran a MM with data from all species combined, allowing random slopes to vary among species and using a standardized measure of age. This model showed that species differed in the association between *D_M_* and age, so we analyzed each species separately. Both *D_M_* and age were centered to 0 and scaled to standard deviation of 1 to facilitate comparison of effects across species. For common marmosets, there was only one repeated observation of an individual, and including a random effect for ID did not improve the model, so we omitted the random effect for this species. We visually checked model residuals and log‐transformed the dependent variable as needed to meet model assumptions.

### 4.4 Mortality and body condition

The data used in survival analyses represent all‐cause mortality (e.g., including death from acute health conditions and humane euthanasia for terminal conditions). We ran Cox PH models with age as the timescale for each species separately. Initial analyses indicated that the interaction between *D_M_* and sex was never significant, so we present results from survival models without the interaction. *D_M_* was centered to 0 and scaled to standard deviation of 1 to facilitate comparison of effects across species. Since we generally had multiple observations per individual, we treated *D_M_* as a time‐dependent covariate and coded each observation as a time interval starting at the age at observation. The time interval ended at the age at the next observation (if there was one), at the age of confirmed death (if there was one), or at 0.25 years after the observation (if there was neither a subsequent observation nor confirmed death). We chose 0.25 years because this was the minimum time between observations in the iPAD database. Each time interval had an associated event outcome, coded as 1 (death) only if it was the last observation for an individual with a confirmed death, and otherwise coded as 0 for earlier observations or unknown outcomes.

For all species of NHPs except cotton‐top tamarins (which lacked sufficient data), we analyzed the relationship between body mass and age. We used body mass as the dependent variable in MMs with fixed effects of age, the second‐order polynomial of age, sex, and the two‐way interaction between age and sex. The exceptions to this were squirrel monkeys and common marmosets, for which we only had body mass data on one sex and hence could not include any sex effects. We included a random intercept for ID and a random slope for age within ID for all species, and we included a random intercept for population if there were multiple populations (chimpanzees and rhesus macaques). Next, we modeled the relationship between *D_M_* and body mass by adding *D_M_* as a fixed effect in the MMs described above. For species with substantial amounts of repeated measurements of body mass within IDs (chimpanzees, rhesus macaques, Coquerel's sifakas, and red‐collared brown lemurs), we also tested whether *D_M_* predicted subsequent change in body mass. We calculated subsequent change in body mass as a rate using mass change between the current observation and the next observation for a given ID divided by time in years, and we used this as the dependent variable in MMs including fixed effects of sex, age, *D_M_*, and the two‐way interactions among them. All models included a random intercept for ID, and the models for chimpanzees and rhesus macaques further included a random intercept for population. We ran Cox PH models for the effect of condition on survival as described for survival models above, substituting mass or change in mass for *D_M_*. We visually checked model residuals and log‐transformed dependent variables as needed to meet model assumptions.

### 4.5 Phylogeny and conservation of homeostasis

We created a matrix of phylogenetic distances based on time since divergence among species from a large recently published phylogeny of living primates (Hedges, Marin, Suleski, Paymer, & Kumar, 2015). We created a matrix of correlations between *D_M_* calculated with each species as a reference for itself and the *D_M_* calculated with every other species as a reference. We then ran a Spearman rank correlation among the cell values of the two matrices, excluding the diagonals (which each represent each species as its own reference). As the matrix of phylogenetic distance is symmetric while the correlation matrix is not, we checked correlations using only the upper or lower half of the matrices. These were also positive and significant and did not change interpretation, so we present the correlation for the overall matrices.

We ran all statistical analyses in the R statistical environment v3.4.3 (R Development Core Team, 2014), with packages “lme4” (Bates, Maechler, Bolker, & Walker, 2015), “survival” (Therneau, 2015), and “coxme” (Therneau, 2015).

## CONFLICT OF INTEREST

None declared.

## AUTHOR CONTRIBUTIONS

G.D., T.W.W., and A.A.C. conceptualized and designed the initial study. G.D. designed and performed the first version of analysis. G.D. and T.W.W. analyzed the data. V.L. aided with analysis. G.D., T.W.W., A.A.C., V.L., J.W.K., and M.A.B. contributed to further development of study design and analysis. J.W.K. and L.F. provided expertise on the data used. T.W.W. wrote the first version and major revisions of the manuscript. All authors read and provided feedback on the manuscript.
