# Supplementary material for: Conservation of physiological dysregulation signatures of aging across primates
Source: Aging Cell. 2019 Feb 11;18(2):e12925. doi: 10.1111/acel.12925 (PMC6413749; doi:10.1111/acel.12925)
Supplement: Supplementary file 1 [file ACEL-18-e12925-s001.docx]

Supplementary Information

Table S1. Physiological biomarkers considered in calculation of dysregulation

| Biomarker (units) | System | CV† |
| --- | --- | --- |
| Hemoglobin (g/dL)* | blood | 0.110 |
| Hematocrit (%) | blood | 0.110 |
| Red blood cells (10^6^/mm^3^)* | blood | 0.194 |
| Mean corpuscular hemoglobin, MCH (pg)* | blood | 0.144 |
| Mean corpuscular hemoglobin concentration, MCHC (g/dL)* | blood | 0.049 |
| Platelets (10^3^/mm^3^) | blood | 0.386 |
| White blood cells (mm^3^)* | immune response | 0.492 |
| Neutrophils (%)* | immune response | 0.247 |
| Lymphocytes (%)* | immune response | 0.389 |
| Monocytes (%) | immune response | 0.678 |
| Calcium (mg/dL)* | electrolytes | 0.069 |
| Sodium (mmol/L) | electrolytes | 0.025 |
| Chloride (mg/dL) | electrolytes | 0.037 |
| Potassium (mmol/L) | electrolytes | 0.136 |
| Cholesterol (mg/dL) | lipids | 0.245 |
| Triglycerides (mg/dL) | lipids | 0.697 |
| Glucose (mg/dL) | metabolism | 0.317 |
| Albumin (g/dL)* | proteins, liver, kidney | 0.129 |
| Alkaline phosphatase (IU/L)* | proteins, liver, kidney | 0.758 |
| Alanine transaminase, ALT (IU/L) | proteins, liver, kidney | 0.787 |
| Aspartate transaminase, AST (IU/L) | proteins, liver, kidney | 0.834 |
| Creatinine (mg/dL)* | proteins, liver, kidney | 0.297 |
| Blood urea nitrogen (mg/dL)/ Creatinine ratio | proteins, liver, kidney | 0.514 |
| Total protein (g/dL)* | proteins, liver, kidney | 0.081 |

* Biomarkers in Set 2, where D_M_ was calculated from the same 12 biomarkers for all species

† Coefficient of variation, calculated from all species values combined

Table S2. Species average *D_M_* values calculated from different samples and reference populations

|  | Set 1 | | Set 2 | |
| --- | --- | --- | --- | --- |
| Focal species | Per-species ref | Common ref | Per-species ref | Common ref |
| Human | 8.471 | 8.867 | 6.075 | 8.344 |
| Chimp | 7.462 | 7.465 | 5.859 | 7.454 |
| Orang | 5.774 | 7.527 | 5.896 | 7.172 |
| Rhesus | 7.991 | 6.326 | 6.667 | 6.674 |
| Pigtail | 7.005 | 4.673 | 6.793 | 8.314 |
| Squirrel | 5.590 | 10.297 | 5.676 | 7.851 |
| Cottontop | 5.258 | 5.391 | -- | -- |
| Marmoset | 11.153 | 7.041 | 6.171 | 9.628 |
| Sifaka | 5.433 | 9.510 | 6.154 | 7.934 |
| Ringtail | 7.199 | 10.957 | 6.030 | 7.783 |
| Rcb | 4.684 | 9.789 | 5.133 | 8.096 |

Table S3. Estimated fixed effects from multilevel models of age and sex effects on *D_M_*

|  | Species | N | IDs | Intercept | Age | Sex | Age x Sex |
| --- | --- | --- | --- | --- | --- | --- | --- |
| 1 | Human | 5933 | 2462 | -0.07 | **0.14 (0.10, 0.19)** | **0.15 (0.09, 0.22)** | 0.02 (-0.04, 0.09) |
|  | Chimp | 2723 | 400 | -0.03 | **0.26 (0.19, 0.32)** | -0.01 (-0.12, 0.11) | **-0.17 (-0.28, -0.05)** |
|  | Orang | 149 | 30 | -0.06 | <0.01 (-0.25, 0.25) | 0.22 (-0.23, 0.66) | **0.55 (0.05, 1.02)** |
|  | Rhesus | 725 | 178 | 0.18 | **0.55 (0.39, 0.70)** | **-0.31 (-0.56, -0.07)** | **-0.30 (-0.49, -0.11)** |
|  | Pigtail | 353 | 92 | 0.01 | **0.26 (0.06, 0.45)** | -0.05 (-0.34, 0.23) | 0.01 (-0.28, 0.30) |
|  | Squirrel | 364 | 26 | -0.03 | **0.24 (0.10, 0.39)** | NA | NA |
|  | Cottontop | 489 | 148 | 0.08 | **0.26 (0.13, 0.39)** | -0.15 (-0.35, 0.06) | -0.01 (-0.18, 0.17) |
|  | Marmoset | 75 | 74 | -0.05 | 0.10 (-0.22, 0.42) | 0.06 (-0.42, 0.54) | 0.15 (-0.34, 0.64) |
|  | Sifaka | 97 | 32 | 0.02 | **0.67 (0.28, 1.06)** | 0.02 (-0.37, 0.41) | -0.44 (-0.88, 0.01) |
|  | Ringtail | 145 | 50 | -0.04 | **0.25 0.03, 0.46)** | 0.08 (-0.24, 0.39) | 0.07 (-0.25, 0.39) |
|  | Rcb | 106 | 25 | 0.03 | **0.61 (0.36, 0.84)** | -0.06 (-0.42, 0.32) | 0.02 (-0.31, 0.34) |
| 2 | Human | 5933 | 2462 | -0.05 | **0.12 (0.07, 0.16)** | **0.13 (0.06, 0.20)** | 0.05 (-0.01, 0.12) |
|  | Chimp | 3129 | 408 | -0.05 | **0.15 (0.08, 0.21)** | 0.12 (-0.01, 0.26) | -0.04 (-0.15, 0.08) |
|  | Orang | 158 | 31 | -0.29 | 0.21 (-0.07, 0.49) | **0.73 (0.23, 1.23)** | **0.77 (0.27, 1.25)** |
|  | Rhesus | 2553 | 202 | -0.14 | **0.35 (0.28, 0.42)** | -0.13 (-0.33, 0.09) | **-0.16 (-0.25, -0.07)** |
|  | Pigtail | 711 | 123 | -0.04 | **0.26 (0.11, 0.41)** | 0.02 (-0.22, 0.27) | -0.14 (-0.36, 0.08) |
|  | Squirrel | 379 | 26 | -0.01 | **0.22 (0.10, 0.35)** | NA | NA |
|  | Marmoset | 83 | 80 | 0.01 | 0.21 (-0.12, 0.55) | -0.01 (-0.47, 0.46) | -0.03 (-0.50, 0.43) |
|  | Sifaka | 101 | 32 | 0.09 | **0.67 (0.28, 1.06**) | -0.10 (-0.49, 0.29) | **-0.46 (-0.90, -0.02)** |
|  | Ringtail | 150 | 50 | -0.20 | 0.05 (-0.18, 0.27) | **0.33 (<0.01, 0.70)** | 0.02 (-0.26, 0.36) |
|  | Rcb | 110 | 25 | -0.16 | **0.35 (0.09, 0.60)** | 0.20 (-0.31, 0.71) | 0.12 (-0.24, 0.48) |

Set 1 uses variable numbers of biomarkers for 11 species; Set 2 uses the same 12 biomarkers for 10 species. *D_M_* and Age were centered to 0 and scaled to 1 standard deviation. 95% confidence intervals are in parentheses. The reference sex is “female”. The intercept reflects the mean biomarker value for females at the median age. Significant effects are in bold. Species are listed from most closely to most distantly related to humans. NA = data not available

Figure S1. Estimated intercepts (averages) and confidence intervals by sex from Table S3, where *D_M_* was centered to 0 and scaled to 1 standard deviation to facilitate comparisons among species.


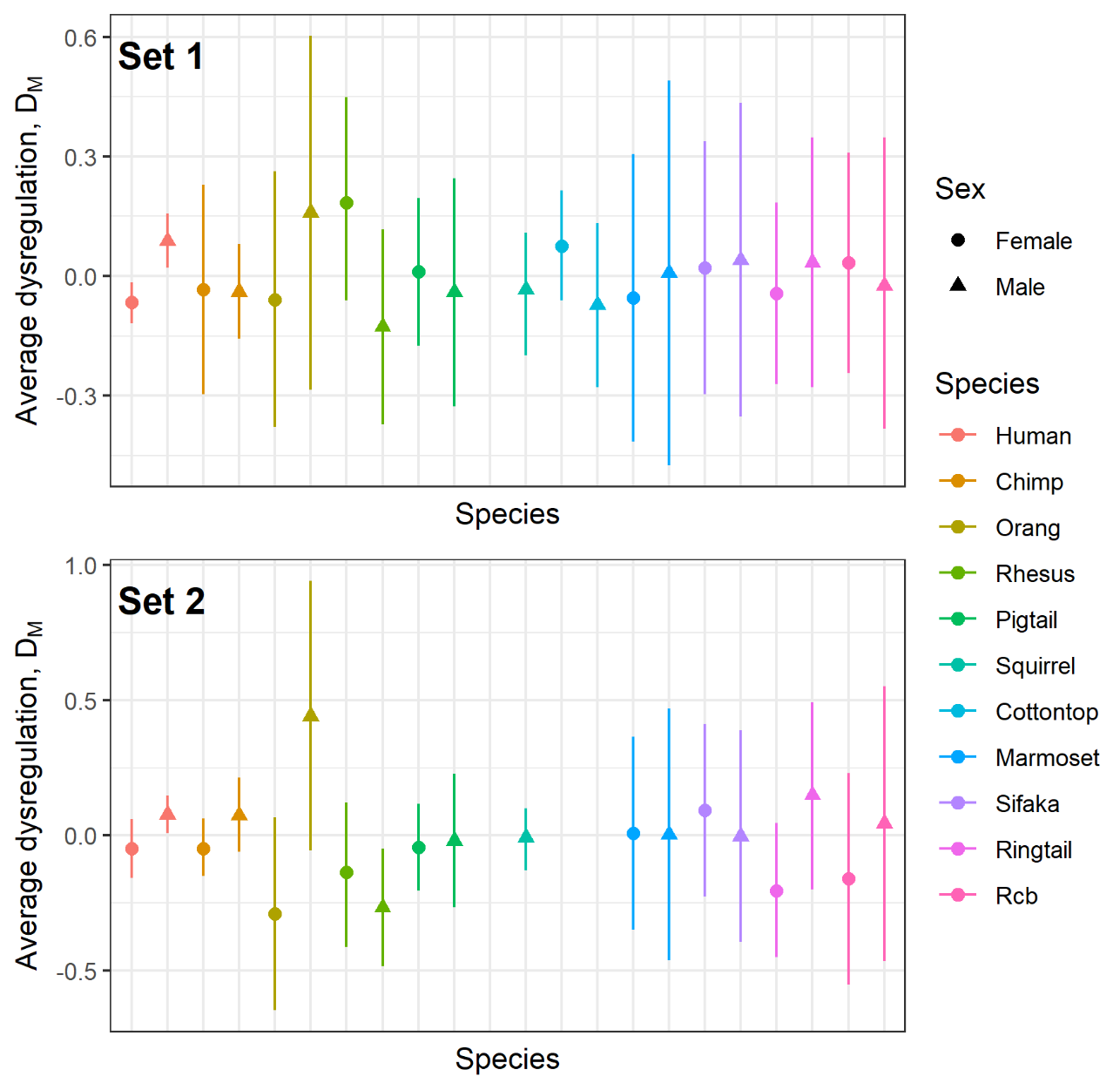


Additional analysis of body mass

To determine if changes in body mass were related to aging in nonhuman primates, we used all available data in iPAD (i.e., including many individuals that were not used in the dysregulation analysis) to model the effects of age and sex on body mass using linear hierarchical models. We included the fixed effects of age, the second-order polynomial of age, sex, and the two-way interaction between age and sex. We included a random intercept for individual ID for all species and a random slope for age within ID for all species except pigtailed macaques. We also included a random intercept for center for chimpanzees and rhesus macaques.

To examine if current body mass or changes in body mass could predict mortality in nonhuman primates, we used data on two species (chimpanzees and rhesus macaques) for which there was ample data on repeated mass measurements per individual and mortality. We calculated recent rate of change in body mass as the current mass minus the previous mass divided by the time (in years) between the two measurements. We ran Cox models with fixed effects of current mass, recent mass change, and sex, and a random intercept for center. We treated D_M_ as a time-dependent covariate and coded each observation as a time interval starting at the age at observation. The time interval ended at the age at the next observation (if there was one), at the age of confirmed death (if there was one), or at 0.25 yrs after the observation (if there was neither a subsequent observation, nor confirmed death). We chose 0.25 yrs because this was the minimum time between samples in the iPAD database. Each time interval had an associated event outcome, coded as 1 (death) only if it was the last observation for an individual with a confirmed death, and otherwise coded as 0 for earlier observations or unknown outcomes.

The linear relationship between age and body mass was usually significantly positive, and there was an extremely consistent significant negative second-order polynomial effect of age in every species (Table S4), indicating that mass initially increased in adults (from younger to middle-aged individuals) but eventually declined (from middle-aged to oldest individuals). In rhesus macaques, common marmosets, and red-collared brown lemurs, lower current mass was associated with higher risk of mortality, whereas the opposite pattern was seen in chimpanzees (Table S5). In chimpanzees, rhesus macaques, and common marmosets, recent weight loss was also increased mortality risk.

Table S4. Estimated fixed effects of age and sex on body mass in adults

| Species | N | IDs | Age | Age^2^ | Sex | Sex x Age |
| --- | --- | --- | --- | --- | --- | --- |
| Chimp* | 5957 | 375 | **0.16 (0.17, 0.20)** | **-0.04 (-0.03, -0.03)** | **0.10 (0.13, 0.21)** | **-0.10 (-0.10, -0.06)** |
| Orang* | 457 | 31 | **0.24 (0.11, 0.38)** | **-0.10 (-0.12, -0.08)** | **0.71 (0.53, 0.88)** | **0.51 (0.31, 0.71)** |
| Rhesus | 29896 | 1079 | **0.43 (0.33, 0.53)** | **-0.47 (-0.49, -0.46)** | **3.89 (3.62, 4.16)** | **0.38 (0.21, 0.54)** |
| Pigtail | 150 | 111 | **1.63 (1.19, 2.07)** | **-0.97 (-1.34, -0.59)** | **2.91 (2.08, 3.74)** | **1.90 (1.15, 2.63)** |
| Marmoset | 2644 | 190 | -0.001 (-0.001, 0.004) | **-0.01 (-0.01, -0.01)** | NA | NA |
| Squirrel | 823 | 26 | 0.02 (-0.01, 0.05) | **-0.05 (-0.06, -0.04)** | NA | NA |
| Sifaka | 854 | 41 | **0.35 (0.09, 0.61)** | **-0.11 (-0.15, -0.07)** | -0.49 (-0.98, 0.01) | -0.02 (-0.34, 0.30) |
| Ringtail | 1588 | 83 | **0.17 (0.08, 0.26)** | **-0.04 (-0.05, -0.02)** | **0.18 (0.05, 0.31)** | -0.04 (-0.16, 0.07) |
| Rcb* | 999 | 36 | **0.05 (0.01, 0.09)** | **-0.02 (-0.03, -0.01)** | **-0.06 (-0.12, -0.002)** | -0.05 (-0.11, 0.001) |

Age was centered to 0 and scaled to 1 standard deviation within species. The reference sex is female. 95% confidence intervals are shown in parentheses. Significant effects are in bold. NA = data not available

* Log-transformed mass

Table S5. Estimated fixed effects from Cox models of the effect of current body mass and change in body mass on risk of mortality

| Species | IDs | Deaths | Current mass HR | Mass change HR | Sex HR |
| --- | --- | --- | --- | --- | --- |
| Chimp | 331 | 71 | **1.85 (1.48, 2.30)** | **0.60 (0.48, 0.73)** | **2.58 (1.47, 4.55)** |
| Rhesus | 912 | 443 | **0.79 (0.69, 0.90)** | **0.64 (0.59, 0.70)** | 1.07 (0.84, 1.38) |
| Marmoset | 178 | 169 | **0.64 (0.54, 0.76)** | **0.78 (0.67, 0.91)** | NA |
| Sifaka | 36 | 13 | 0.55 (0.22, 1.39) | 0.91 (0.52, 1.58) | **0.17 (0.04, 0.74)** |
| Rcb | 35 | 14 | **0.38 (0.17, 0.87)** | 0.50 (0.22, 1.14) | 1.04 (0.30, 3.56) |

Current body mass and change in mass were centered to 0 and scaled to 1 standard deviation, within species. The reference sex is female. Significant effects are in bold. HR = hazards ratio; NA = data not available
